# Extra Supplement—The Nursing Mirror

**Published:** 1890-07-05

**Authors:** 


Being the Extra Nursing Supplement of "The Hospital" Newspaper.

All Contributions for this Supplement should be addressed to the Editor, The Hospital, 140, Strand, London, W.C., and should have the word "Nursing" plainly written in left-hand top corner of the envelope.

### En Passant.

SYDENHAM NURSES.—The eighth annual report of the Sydenham Nursing Association is out, and tells of an income of £74 and a balance of £15 in hand. Sister Ruth is the working member of the society, and her services seem to be gratefully appreciated by the poor whom she tends. Drugs have been sold at cost price, surgical appliances have been lent or given, hospital letters have been obtained, and all done for the sick of Sydenham, which sympathy could suggest.

SHORT ITEMS.—Lady Cadogan signs an appeal for funds for the Pimlico District Nursing Association.—Miss Heanley, of Boston, is back at work; Miss Spencer, of Edinburgh, is still away, but is much better.—Princess Louise will open the bazaar to be held in Knole Park on July 15th and 16th in aid of the Kent Nursing Institution.—Mrs. Newcomb, aged 74, is still living in New York. She began her nursing career as an Army Sister at Cairo, in 1862, and was afterwards matron of Vicksburg Hospital, and nursed in Tennessee during the Civil War.—Miss Lamb, after five years' good work, has resigned her post as matron of Taunton Hospital.

IN MEMORY OF THE LATE MISS FISHER.—A brass memorial tablet, bearing these words, has been erected at Philadelphia: "Alice Fisher, born at Queen's House, Greenwich, June 14, 1839. Died at this hospital June 3, 1888. Chief nurse of the Philadelphia Hospital Training School for Nurses, from November, 1884, until her death. By her knowledge, capacity, and fitness for the place, and, above all, by her devotion and her personal character, this training school for nurses was established." The benefits that have resulted to the hospital, to its patients, and to the community are the works that follow her. This tablet has been placed beside the picture of Miss Fisher in the reception-room at "Blockley." It is the gift of some friends of the late lamented lady.

BABY FARMING.—A disgraceful case of baby farming has come to light in Dublin, in which, unfortunately, a hospital nurse is implicated. An inquest was held on a child which had been starved to death, and a juror stated that it was rumoured that the nurse who placed the infant in the hands of the baby farmer got £40 for the transaction. Now we cannot but hope that this nurse acted in ignorance; surely no woman could deliberately hand over a tender babe to a lingering and cruel death. Baby farming is a shame to this century, and we earnestly beg all nurses, no matter how tempted, to steadily refuse to further any transaction which removes a child from its mother. Specious advertisements of ladies who desire to adopt children—always with premium—are to be regarded with suspicion. In one case lately brought to light fourteen infants had been received, and twelve had died. A verdict of manslaughter was obtained in this case. The picture exposed of these poor neglected wee babies slowly dying the most cruel and bitter death, was enough to make the hardest heart bleed. Once for all, baby-farming is another word for murder; no shame could be too great to heap on a nurse who aided in any way this nefarious practice.

HALIFAX FEVER HOSPITAL.—Our paragraph on the nursing of this hospital has had the result of starting inquiries by a resident in Halifax, who gives his experiences in a local paper: "During the last fortnight or so—in fact, since the appearance of the paragraph quoted above—I have made pretty thorough and complete investigation of facts as they now appear; and the result has been unmistakably to strengthen and confirm me in the view I have been accustomed to take from the establishment of the place—which view I have already indicated after the plainest possible fashion." It is so evident that Halifax means to have one of the best infirmaries in the land, it would be a terrible pity to leave the blot of an imperfect fever hospital to spoil the picture. At least, let there be a thoroughly trained nurse in charge of the fever patients.

SHEFFIELD NURSES' HOME.—The annual meeting was held on June 17th, and Mr. Richardson presided and made an able speech. He pointed out that it appeared to him to be unfortunate that whilst the receipts from the nursing amounted to £960, the expenditure, notwithstanding it had been greatly reduced, reached £1,226. After the length of time which the institution had been established the receipts, he thought, ought to equal the expenditure. He did not say this because he wished to see the subscriptions reduced, as he should be sorry if such were the case, but there was the concluding paragraph of the report, which said that the institution was established to undertake a branch work, which it had not yet seen its way clear to adopt, namely, the gratuitous nursing amongst the poor. We await the arrival of the report of this Home before commenting further on this discrepancy between receipts and expenditure, in what is generally regarded as a lucrative undertaking.

THRIFT IN PHILADELPHIA.—We have received the by-laws of the Nurses' Beneficial Association of Philadelphia, and are very much struck by the business-like look of this small book. Apparently the Board of Managers consists of women only, and yet the constitution, the benefits, the order of business at the annual meetings, &c., are all expressed not only in plain but in legal language. Have they some lady lawyer amongst their friends, these energetic American nurses? Each member of the Association pays in fifty cents a month, and in return she is entitled to fifty dollars on her death (this is cheerful!), and also, according to Section I., to the following benefits in sickness: "Any member unable, by reason of sickness or accident, to attend to his or her duties as nurse, shall notify the Secretary in writing within three days after the occurrence of the disabling cause, stating the nature thereof and furnishing his or her name and residence. He or she shall also furnish a certificate from the physician in attendance, which shall be made out on a blank furnished by the Secretary for that purpose. The Secretary shall then notify the Visiting Committee, and upon their favourable report, the member shall be entitled to receive five dollars per week, payable weekly, no allowance to be paid for less than one week's illness, nor for more than twelve weeks in any one year, the year to date from the first week's benefit." We wish this vigorous young society all success. It is always a pleasure to us to see how independent and thrifty our American cousins are; it augurs well for the future of our English nurses; gradually they will attain breadth of mind and greater business capabilities.

### Notes from Australia.

(*By Our Own Correspondent.*)

MELBOURNE, MAY 1st.

WE have been having a Royal Commission on Charities here, and of course hospitals and nursing have afforded much evidence. Captain Evans, Inspector of Public Charities, stated that it would be wise if the system of female nursing were introduced more generally into the hospitals of the colonies. It would be a great benefit to the hospitals and the public if there were a large number of trained nurses. Nurses should be treated as persons who had taken up a profession, and wished to learn. They should dine apart from the men employed in the same institutions. Nurses should also have separate rooms. Captain Evans also said it was very necessary that nurses should be certificated. The Committee and medical staff could form the standard of efficiency. But there should be one uniform standard. Schools for training could be found in the existing hospitals. A trained nurse, independent of her nursing duties, should not be called upon to do rough domestic work. Nursing opened a wide field to women. He preferred the title of Lady Superior to that of Matron, as giving the head of an institution a better position. Periodical examinations of the nurses should be held, and there should be a central board of examination. Of course Captain Evans had tales to tell of many miserable impostors who live on charity, but he highly commended the work of the Salvation Army, the Women's Benevolent Societies, and the Charity Organisation Society. With regard to the last, Professor Morris has just come back from Brooklyn with a good story: "I asked Mr. Buzelle, who, in his opinion, were the chief authorities on the subject of organisation in charity. 'St. Paul and Octavia Hill,' was his striking reply. 'That's a high compliment to Miss Hill,' I said, 'but about St. Paul? "If a man will not work, neither shall he eat." I suppose that's what you mean.' 'That partly,' he replied, 'but rather, "though I give all my goods to feed the poor and have not charity."'"

The District Nursing Society continues its good work. Miss Eva Hurnall has entered its service as nurse, in place of Miss Cannon, resigned. The uniform has been altered from grey to black at the request of the nurses. The Society has a balance of £200 in hand. Typhoid and diphtheria are not quite so virulent as they were last year, still our percentage of deaths from infectious diseases is far too high, and would shock you English hospital workers. The Board of Public Health has just decided to prosecute certain medical practitioners for neglecting to report infectious cases; they have also severely censured the Shire Council of Numurbrah for neglecting to require their medical officer to visit a district where diphtheria was epidemic. I suppose in time, by these and other measures, we shall have fewer preventible deaths, and in the meanwhile we must remember that Rome was not built in a day.

There is a proposal to the fore just now to build a foundling hospital; this would be, in one sense, a benefit to the colony, as each infant represents a unit assessed at £100 value to the State. Whether it is wise to take from an unfortunate mother the responsibility she has brought on herself is another question. Still, infanticide is terribly on the increase in Melbourne.

The Committee of Geelong Hospital has been called upon to resign. A meeting was held at the Town Hall, and Dr. Deane spoke. He referred to the sudden dismissal of trained nurses, and moved: "That in consequence of the recent revelations in the management of the Geelong Hospital, which are seriously detrimental to its well-being, this meeting is of opinion that a thorough reform in its government is required, and that such reform can be best brought about by the resignation of the present committee." Councillor E. J. Jones seconded the resolution. He protested against the action of the present Committee in raising the salary of the resident surgeon to £300, and the salary of the Superintendent £25 per annum. He objected to their excessive expenditure, and thought that they had acted illegally, and therefore they had forfeited his confidence. If a new committee were elected, he would vote for some but not all of the present members. He thought there were too many professional men on the board, and they wanted more business men. The resolution was carried, and it remains to be seen whether the needed reforms will be carried out.

Melbourne Hospital has just received a legacy of £12,000. The new Matron is doing good work, but it will be long before the nursing system is in thorough order. Young nurses cannot work well for fourteen hours a-day. Miss Martelli is much missed at the Sick Children's Hospital, and her successor has been succeeded again. Still, we are progressing, and nursing daily becomes a more important and popular subject.

### The Nurses' Bookshelf.

### THE TRAINING OF NURSES IN FRANCE.*

THE vexed question in the nursing world in France, is, and has been for years, the comparative merits of lay nurses and of religious Sisters. We are constantly hearing that the present lay nurses are not satisfactory and that many long for the old days when the *sœurs de charité* reigned in the wards. As usual there are two sides to the question; in favour of the past system it can be said that the Sisters were extremely kind to the patients, and ever true to their duty; and that the present nurses are known to be more worldly and some of them have been known to accept bribes. But, on the other side, the Sisters were more devoted to priests than to doctors, they trained their probationers in theology more than in nursing, and they produced that discordant element which must ever be where there is a divided duty. The Sisters got behindhand in scientific knowledge, they failed to fulfil all that was demanded of the doctor's handmaid, and therefore they lost their power. The present nurses are not all that is to be desired, we admit, but then no system is perfection at first, and these present nurses are only gradually being trained up to the high standard the doctors desire. Those who read the manuals issued to nurses by the *Progrès Medical* cannot but acknowledge that if the probationers now do not learn, it is their own fault; the doctors are doing their best to train them at all events, as the series of handbooks we are now reviewing absolutely proves. The first of the series is on Anatomy and Physiology and is illustrated with twenty-seven well-drawn figures, far better than we have seen in any English book on nursing. This volume has been edited by M. H. Duret and M. P. Regnard, and last year reached a fourth edition; this is a fact to be noted by those who insist that nursing in France does not become more scientific. The book begins with an account of the formation of nursing schools at Bicêtre, at the Salpêtrière, and at La Pitié, and gives the following list of subjects in which a nurse has to pass ere she secure her diploma: Anatomy, Physiology, Surgical Dressing, Hospital Management, Hygiene, Pharmacy, Monthly nursing and practical bandaging. Starting the nurse towards obtaining the knowledge necessary to pass this examination, the first chapter opens with a general description of the human body, the skeleton coming at once under observation. Several pages and two illustrations are given to the consideration of the skull; its state at birth; the names and positions of the different bones, &c.; then the trunk and limbs are

* Mannel Pratique de la Garde-Malade et de L'infirmière publié par le Dr. Bourneville. (Paris: Bureaux du Progès Medical.)

separately considered. The third chapter deals with the muscles, and deals with them very freely too, some excellent figures illustrating the letter-press. Strangely enough, an English nurse is seldom taught aught about the muscles of the body, her anatomy generally stops short with the bones. The heart and circulation are dealt with in the fourth chapter, the course of the principal arteries and veins being traced with care. The nervous system is discussed under three heads, the brain, the nerves, and the organs of sense. An article on the viscera brings the study of anatomy to a close, and we commence on Physiology. Be it at once confessed that there is a certain frankness in these French books which is startling even when we remember they are addressed to professional nurses and to mothers of families; more especially notable is this in a somewhat sentimental chapter on the stages of life, which is not intended to be scientific and so has no excuse for the absence of reserve. Nor do we admire the philosophy which gives all the joys of life to youth, and which regards old age as "the most terrible stage of life." Better, far better, is Browning's—

"Grow old along with me,

The best is yet to be,

The last of life, for which the first was made.

Our times are in His hand,

Who said 'A whole I planned.'

Youth shows but half, see all, trust God, be not afraid."

But we must give the concluding paragraph of this volume on Anatomy and Physiology, before we turn next week to consider the second volume of the series: "Here, then, are shown the principal points of interest in the study of the human body. Do not think that you now understand anatomy and physiology, you are simply able to better comprehend the doctor's orders with regard to the patients under your care. But another advantage you should have gained from these studies is a true appreciation of the human body wherein the least organ exceeds in beauty the most perfect machine, and whose functions are all so marvellously adjusted. It is in such contemplation our minds can best gain healthy satisfaction, and the most pure enthusiasm."

*(To be continued.)*

### Everybody's Opinion.

*[Correspondence on all subjects is invited, but we cannot in any way be responsible for the opinions expressed by our correspondents. No communications can be entertained if the name and address of the correspondent is not given, or unless one side of the paper only be written on.]*

### LADY WORKERS WANTED.

The Hon. Secretary of the Zenana Medical College, St. George's Road, S.W., writes: We were attracted the other day by a paragraph in one of the daily papers referring to some charitable agency, and headed "Lady Workers Wanted," and we felt that we, too, would re-echo the cry, and say "Lady Workers Wanted." We, too, would feel encouraged by volunteers in the service. Allow me to suggest some ways in which valuable help might be rendered:—(1) Ladies could visit the college, and hold Bible readings with our students. (2) Ladies could help our maternity department by sending cast-off garments for poor mothers and infants; dinner, and soup, and coal, and convalescent hospital tickets. (3) Many might take cards, books, and boxes, and collect for our funds for giving free or partially free education, board, and residence to candidates unable to pay for their own training. If friends could only see our book filled with application letters, which we have to lay aside for want of funds, they would realize how valuable such help would be. (4) Ladies might become hon. local secretaries in different districts, and scatter reports and papers among their friends and neighbours, and thus make the work known. (5) Ladies clever with their fingers might make pretty and useful articles for sale on our behalf. Many would be interested and much might be done. We would suggest that ladies disposed for such work should kindly send in their names. A working party could at once be established at the college. In any of the above-mentioned ways help will be greatly appreciated.

P.S.—The college is now full.

### A HOME OF REST FOR NURSES.

Miss Louisa Twining writes: As a correspondence is still being carried on in your columns upon this subject, I venture to add one or two remarks which have occurred to me with regard to this movement. I read of the various suggestions made at the recent meeting, with regret that so influential a company should set forth so extensive and expensive a scheme while many more important movements are on foot. Surely we cannot help feeling that there is at the present moment *trop de zèle* on behalf of nurses, and that at least some of the sympathy is misapplied. Why should nurses, taken mainly from the servant class, require such special efforts to be made for their holidays, any more than these persons, and we have not yet heard it considered necessary to provide homes for our domestics. Why have not nurses families and friends to visit during their brief vacations as well as our children's nurses, our cooks, and our housekeepers? and, if so, it is natural to suppose that their time, rest, and recreation would be found in such families, and not in a continuation of institution life, for "homes" must be carried on with strict rules and regulations, and nurses, like all other people, need to be taken out of their grooves and surroundings when "off work." After some years' experience of about fifty nurses in a large infirmary, I do not remember any who required to be helped in their holidays. Even if a room had to be taken near friends, a part of wages had better be thus spent than in a too often lavish outlay on dress. And surely for the few there may be who are wholly without families and friends there are already homes of rest to be found, which would be better than any special ones could be. I earnestly hope that the large sum of £15,000 asked for may be devoted to more urgent needs than this. Let me add how cordially I agree with the suggestion that three weeks instead of a fortnight should be the term of a holiday, not only for nurses, but for parochial mission women and others engaged in trying work with the sick and poor. I have frequently been told that the first week is spent in resting from the effects of a change or a journey, and in the second week the benefit begins to be felt, and then the return to work follow all too quickly for permanent results to be gained.

### FEVER NURSES.

Nurse Laura writes: I was very pleased to see someone had at last spoken a word in praise of fever nurses. I think, considering the noble, self-sacrificing life they lead, they certainly deserve the highest commendation. Many are even prevented from visiting their own relatives (where there are children people naturally fear infection), and yet they cheerfully take up their cross, and faithfully perform the many painful duties devolving upon them. Surely they do indeed deserve the praise of all, and we know that our Father in heaven looks down upon this work of love, and gives them the daily strength they so much need. They have their reward in knowing—

That to comfort and to bless,
To find a balm for woe,
To tend the lone and fatherless,
Is angels' work below.

And we believe thy Word,
Though dim our faith may be,
Whate'er for Thine we do, O Lord,
We do it unto Thee.

### INFANTS AND CASTOR-OIL.

A District Midwife writes: I have cut the enclosed from the *People* for June 15th. It seems to me to require explanation. Castor-oil has hitherto been regarded as the safest medicine one could give small babies, and I, as a midwife of many years' standing (trained at Queen Charlotte's, and holding the Obstetrical Society diploma), should not hesitate to give it, if the state of the child seemed to call for an aperient.

Nor have I ever before heard of castor-oil causing either congestion of the brain or convulsions. Mr. Braxton Hicks is not a medical man, though the son of an eminent one; and if, as he says, midwives are not to give castor-oil in future, I should like to know what is to be its substitute. It would be satisfactory to know if Mrs. Mitchell is a qualified midwife; also the dose of oil she gave, and for what purpose. I, not many years ago, heard the matron of a hospital give a lecture on nursing, in which she said the way to give castor-oil to young children was to put "a little brandy in a glass, then pour in the oil, and add a little peppermint water," and if Mrs. Mitchell proceeded according to this method (I should say that no quantities were given) I should not be surprised at the child dying. But I cannot believe that a properly administered dose, say ½ a teaspoonful could cause death. The following is the cutting referred to:—"Mr. A. Braxton Hicks held an inquest at the Local Board Offices, Wimbledon, on the body of Percy Gundry, aged four days, son of a labourer, living at 28, South Road, Wimbledon, who had been found dead in bed. The father stated that his wife was attended by a Mrs. Mitchell, and the child went on very well for the first day or two, but then seemed rather unwell. At seven o'clock on the morning in question, his wife found the little one dead by her side. During the night it had been very restless. Mrs. Mitchell said that as the baby appeared to be not so well she gave him a dose of castor oil. Dr. Walker stated that this was a most injudicious act on the part of the midwife and in his opinion gave rise to congestion of the brain producing convulsions, the cause of death. The Coroner warned Mrs. Mitchell against resorting to castor oil in future, and she replied that she had used it for fifteen years without anything happening. The Coroner told her that something had happened at last."

### A SEASIDE HOLIDAY.

A. E. P. writes:—"I have just been spending a very pleasant holiday at Sharnhen House, Kingsnorth Gardens, Folkestone, a Home of Rest for Nurses and Professional Gentlewomen. I feel sure there are many nurses who would be glad to hear of such a home at this time of year. There are no rules whatever, the visitors do not even make their own beds. The food is excellent and abundant, and the aim of the lady in charge seems to be to make her visitors feel thoroughly at home and really find there the rest and perfect freedom that we so greatly need. If you will kindly insert this in The Hospital I shall be glad, as I feel so thankful myself at having spent such a pleasant holiday that I should like others to know of this home of rest too."

### Presentations.

Mrs. Hatton has been delivering an interesting course of lectures on "Sick Nursing" at Sevenoaks, and after the last was presented with £5 by the ladies' committee, to be given *to any charity she chose. The women who had attended a similar course at the Lay Association Mission Room presented* Mrs. Hatton with a gold pencil-case.

Miss F. Simpson and Miss A. Parnaby, of York County Hospital, have been presented with prizes for their efficiency *in physiology.*

### Notes and Queries.

*To Correspondents.—1. Questions may be written on post-cards. 2. Advertisements in disguise are inadmissible.* 3. In answering a query please quote the number. 4. If a private answer is desired, a stamped addressed envelope must be forwarded.

Notice.—We must really ask our correspondents to be more particular in enclosing name and address. Constantly we receive queries or letters *with no name attached.*

### Queries.

(24) Dispensing.—Where can I learn dispensing in return for my services as a nurse?—*Mary.*

### Answers.

*Nurse Hilda.*—*The Stockholm hospitals are mainly supplied with nurses by the Diakonin Institute.* So far as we know, *foreign nurses* are not received. Perhaps some of our correspondents can tell you more about nursing at Stockholm.

*Sigma.*—We have no room to open up this vexed question again.

(16) *Private Cases Wanted.*—Try Miss Hooper's Agency, 9, Upper Baker Street.

(18) *Encyclopædia.*—Advertise in the sale and exchange column of The Hospital and you will probably get a cheap second-hand copy.

(19) *Canary Isles.*—Write to the secretary of the Canary Islands Company, 1, Laurence Pountney Hill, London, E.C., for information. There is a trained English nurse there, but I should think more would soon be wanted.—*Invalid.*

*** Correspondents whose communications do not appear in the current number are requested to refer to the following week's issue.

### Vacancies.

The following vacancies are announced:

### Matrons.

Paddington Infirmary: £30.

Sanatorium Clergy Daughters' School; £30.

### Nurses.

Buckingham Nursing Home; £16.
Barony Parish Hospital; £24.
Barnstaple Nurses' Home; £25.
Brixton Institute.
Burton-on-Trent Institute.
Blackheath Institution.
Blackburn Infirmary; £27.
Cheltenham Hospital.
Croydon General Hospital; £25.
Cumberland Infirmary, Carlisle (night); £24.
Dumfries and Galloway Infirmary; £21.
Edinburgh Longmore Hospital; £20.
Frome Nurses' Home.
Glamorgan Infirmary; £24.
Greenwich Union; £20.
Greenwich Union (Head); £27.
Greenwich Union (night); £30.
Home for Epileptics, Liverpool; £20.
Highbury Institute.
Ipswich Nurses' Home; £22.
Leicester Fever Hospital; £25.
Manchester and Salford Sick Poor Institution; £25.
Margate Sanatorium; £24.
North-Western Fever Hospital; £36.
Newcastle Infection Hospital; £30.
Paris Levick Institution.
Scarborough Institute.
Sheffield Nurses' Home.
South-Western Fever Hospital; £30.
Stockton Nurses' Home.
Shoreditch Infirmary (temp.); £22.
Weston-super-Mare Institute.
Workhouse Infirmary Nursing Association.
West Cornwall Infirmary, Penzance; £16.
Western Fever Hospital; £30.

### District Nurses.

Bristol District Nurses' Society.
Birmingham Nursing Society.
Hulme Nurses' Home.
South Wimbledon; £100.

### Probationers.

Brompton Consumption Hospital.

Bedford Infirmary.

Central Ophthalmic Hospital.

Children's Convalescent Home, West Kirby; £10.

New Hospital for Women, N.W.

St. Helens Cottage Hospital.

Wrexham Infirmary.

Workhouse Infirmary Nursing Association.

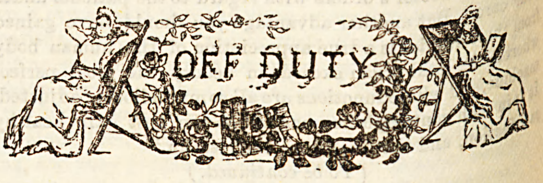

### Amusements and Relaxation.

### SECOND QUARTERLY WORD COMPETITION.

Commences July 5th, 1890, ends Sept. 27th, 1890.

Proper names, abbreviations, foreign words, words of less than four letters, and repetitions are barred; plurals, and past and present participles of verbs, are allowed. Nuttall's *Standard* dictionary only to be used.

*N.B.—Word dissections must be sent in WEEKLY to 140, Strand,* W.C., arranged alphabetically, with correct total affixed.

The word for dissection for this, the FIRST week of the quarter, being

"THUNDER."

| Names. | June 26th. | Totals. |
|---|---|---|
| Adeline | — | 149 |
| Patience | 162 | 622 |
| Lightowlers | — | 335 |
| Canary | — | 86 |
| M. E. S. | — | 37 |
| *Ambition* | — | *36* |
| *M. W.* | — | *217* |
| Esperance | — | 218 |
| Judy | — | 37 |
| Reldas | 153 | 608 |
| Merenda | — | 29 |
| Lucy Locket | — | 40 |
| *Camellia* | — | *30* |
| *Qu'appelle* | *142* | 571 |
| Tinie | 164 | 627 |
| Nurse Mildred | — | 190 |
| Coralie | 155 | 601 |
| Gladys | — | 409 |
| Agamemnon | 158 | 618 |
| G. E. J. C. | — | 34 |
| S. E. A. | — | 24 |
| Jenny Wren | 143 | 559 |
| Multum in parvo | — | 32 |
| *Ecila* | — | 39 |
| Weta | — | 259 |
| Henri | — | 161 |
| Holland | 156 | 611 |
| T. J. | — | 111 |
| Nurse Isabel | — | 17 |
| Nurse Faith | — | 9 |
| *E. S. Z.* | — | *10* |
| *G. P.* | — | *10* |
| Stumps | — | *33* |
| Bolton | — | 8 |
| Jesmur | — | 53 |
| Ran | — | 17 |

### Notice to Correspondents.

N.B.—Each paper must be signed by the author with his or her real name and address. A *nom de plume* may be added if the writer does not desire to be referred to by us by his real name. In the case of all prize-winners, however, the real name and address will be published.

Competitors can enter for *all* quarterly competitions, but no competitor may take more than *one first prize* during the year.

